# Genetic Diversity and Population Structure of Natural *Lycorma delicatula* (White) (Hemiptera: Fulgoridea) Populations in China as Revealed by Microsatellite and Mitochondrial Markers

**DOI:** 10.3390/insects10100312

**Published:** 2019-09-23

**Authors:** Li Zhang, Wenhui Zhao, Fuping Wang, Daozheng Qin

**Affiliations:** 1 Key Laboratory of Plant Protection Resources and Pest Management of the Ministry of Education, Entomological Museum, Northwest A & F University, Yangling 712100, Shaanxi, China; zhangli0927@aliyun.com; 2 Plant Quarantine Station of Plant Protection, Kaifeng 475000, Henan, China; zhaowenhui15@163.com; 3 Yangling Xianglin Agriculture Biotechnology Company Limited, No. 8 Binhe Road, Yangling 712100, Shaanxi, China; fpw616@126.com

**Keywords:** spotted lanternfly, Hemiptera, forestry pest, population genetic structure, microsatellite, *ND2*, *ND6*

## Abstract

The spotted lanternfly, *Lycorma delicatula* (White) (Hemiptera: Fulgoridae), is a polyphagous pest originating in China and now widely distributed in Asian countries. This is one of the more serious forestry pests with a broad host range and causes significant economic losses. Molecular comparison has been used to investigate this pest’s origin in China, and recent studies have explored the genetic structure among populations in Korea. However, the population structure of this pest in China remains poorly understood. In this study, 13 microsatellite markers and two mitochondrial markers (from nicotinamide adenine dinucleotid (NADH) dehydrogenase subunit 2 (*ND2*) and NADH dehydrogenase subunit 6 (*ND6*) regions) were used to reveal the origins and dispersal of *L. delicatula* based on a genetic analysis of Chinese populations from eight locations. Results show a low to high level of genetic differentiation among populations and significant genetic differentiation between both two clusters and four clusters. The network and phylogenetic analyses for mitochondrial haplotypes and population structure analyses for microsatellite datasets suggest that there is potential gene flow between geographical populations. The populations from Zhejiang and Fujian provinces may come from the other geographical populations in north China. The populations from Beijing, Henan, and Anhui provinces were regarded as the major source of migrants with a high number of migrants leaving (the effective number of migrants (*Nem*) = 24.40) and the low number of migrants entering (*Nem* = 2.05) based on the microsatellite dataset, where significant asymmetrical effective migrants to the other populations were detected by non-overlapping 95% confidence intervals.

## 1. Introduction

The spotted lanternfly, *Lycorma delicatula* [1] (Hemiptera: Auchenorrhyncha: Fulgoridae), is a polyphagous pest first found in north China [1,2,3]. It is now not only distributed widely in many Asian countries, including Bangladesh, India [4,5,6,7], Vietnam [8], Korea, and Japan [9,10], but has also been detected as an invasive insect species in North America [11,12,13,14]. *L. delicatula* is one of the serious forestry pests with a broad host range including fruit trees and ornamental plants [11,15,16]. It is univoltine, with nymphs emerging from April to May, becoming adults from late June to August, and laying eggs from August until late November [5,17,18]. Both adults and nymphs can pierce and suck the phloem sap of young leaves and tender shoots, causing plants to wilt and branches to become deformed [19,20,21]. A sooty mold disease that interferes with photosynthesis will result due to the overall damage and the sugary excretions of *L. delicatula* [17,22]. For example, this pest pierces and sucks the branches and leaves of grapevines, leading to a decline in the quality and yield of grapes, and has resulted in extensive economic losses in western Korea (such as Seoul, Incheon, Cheongju, Cheon-an) [18,23].

In the recent past, some Korean researchers had recognized the economic importance of this pest and tried to explore the genetic structure and level of genetic diversity among populations of this pest. Twenty-three microsatellite markers and two mitochondrial markers had been developed for detecting the population structure and origin of *L. delicatula* [24,25]. Seven microsatellite markers have been used to explore the gene flow and dispersal patterns of *L. delicatula* in nine locations in Korea; results indicated a recent range expansion and complex dispersal patterns [26]. A molecular comparison based on two mitochondrial markers (*ND2* and *ND6*) suggested that *L. delicatula* in Korea and Japan might have originated from areas north of the Yangtze River in China [10].

*Lycorma delicatula* have been reported as a drug in Chinese medicine dating back to the 1100s CE [2,3,18]. This pest is now distributed widely throughout China due to its polyphagy, its broad array of host plant species (some plants having broad geographic ranges, such as grapes), and increased winter temperatures [13,27,28]. However, what is the relationship between populations in China? In addition, little is known about the genetic structure, gene flow, and patterns of dispersal of this pest in China. In this study, eight geographical populations in China were selected to elucidate the population structure, genetic differentiation, and relationships between *L. delicatula* populations based on microsatellite and mtDNA datasets.

## 2. Materials and Methods

### 2.1. Sample Collection and Experimental Analysis

**Figure 1:**
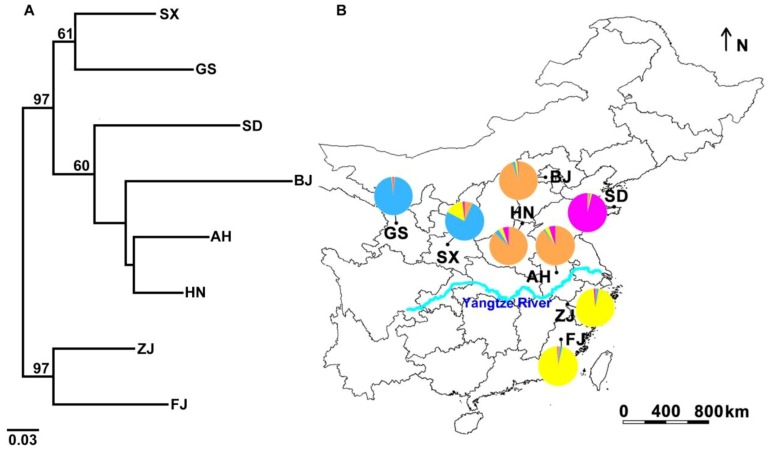
The distribution of eight *Lycorma delicatula* populations in China and the population structure analysis based on microsatellite markers. (**A**) The neighbor-joining tree was re-sampled with 1000 bootstraps, and the bootstrap values of more than 60 are given. (**B**) Population abbreviations are shown in Table 1; the proportion of populations from four clusters inferred by STRUCTURE analysis is shown in the pie charts; the map shows the distribution of the samples.The base map was obtained from URL: http://www.diva-gis.org/gdata. The brilliant blue line represents the Yangtze River.

Specimens of *L. delicatula* were collected from 8 localities in China (Figure 1 and Table 1) using a five-point sampling method (five plots from each locality). Five to 10 samples were obtained from each plot (more than 100 square meters), with plots spaced a minimum of 10 m apart. A total of 183 individuals were collected from *Ailanthus altissima* Swingle. All samples were preserved in absolute alcohol at −20 °C until they were identified and used for DNA extraction. They are now deposited in the Entomological Museum, Northwest A and F University (NWAFU), Yangling, China.

Total genomic DNA was extracted from each adult individual using the DNeasy Blood and Tissue Kit (QIAGEN, Hilden, Germany). DNA concentration was measured using an ND-1000 spectrophotometer (Bio-Rad, Hercules, CA, USA) and diluted to 20 ng/μL with ddH_2_O.

Twenty-three microsatellite markers were used in amplification for 183 individuals of *L. delicatula*. Thirteen of those, with successful amplification and polymorphism in 80% of the specimens, were retained for analysis (Supplementary Material, Table S1). Microsatellite markers were fluorescently labeled (in the 5′ end of forward primers) and amplified in 10 μL total volume with 20 ng DNA and containing 2 mM MgCl_2_, 250 μM dNTP, 1 μM each of forward and reverse primers and 0.1 U of Taq DNA polymerase (TaKaRa, Dalian, China). The polymerase chain reaction (PCR) program consisted of an initial denaturation step of 5 min at 95 °C, followed by 35 cycles of 30 s at 95 °C, 40 s at 54–61 °C (annealing temperature of primers in Table S1), and 30 s at 72 °C, and a final step of 5 min at 72 °C. Electrophoresis of the amplification products was conducted in a capillary sequencer ABI 3730xl (Applied Biosystems, Foster City, CA, USA), and the resulting chromatograms, with GeneScan LIZ-500 size standard were analyzed using GeneMapper v4.1 (Applied Biosystems, Foster City, CA, USA). The allele sizes were manually checked and scored based on trace data for further analysis.

The fragments of mitochondrial NADH dehydrogenase subunit 2 (*ND2*) and NADH dehydrogenase subunit 6 (*ND6*) were amplified using primer ND2-238F (5′-AATTGCCCCATTAATGAAAGA-3′), ND2-866R (5′-TTTGATTTGGTTATTGTA GGGATT-3′) and ND6-87F (5′-TCAAACAGCCTTAATGTGCAG-3′), ND6-480R (5′-TGGTCCTTCAAATGTTCTTACG-3′) [10]. PCR was carried out in 25 μL volumes, including 20 ng DNA, 1.5 mM MgCl_2_, 200 μM dNTP, 0.4 μM each of forward and reverse primers and 0.2 U of Taq DNA polymerase (TaKaRa, Dalian, China). The thermal cycling profile was 95 °C for 5 min, followed by 34 cycles consisting of denaturation at 95 °C for 30 s, annealing temperatures at 58 °C for 40 s, an extension at 72 °C for 30 s. An additional extension ran at 72 °C for 10 min. PCR products were examined with 1% agarose gel electrophoresis to confirm amplification success. The amplification products were sequenced using the ABI 3730 automated sequencer (Applied Biosystems, Foster City, CA, USA).

### 2.2. Genetic Diversity Analyses

Micro-checker was used to check stuttering, large allele dropout, and the sign of the null allele [29], and FreeNA was used to evaluate the frequency of null alleles with the exception maximization (EM) algorithm [30]. The number of alleles (Na), the number of effective alleles (Ne), the observed and expected heterozygosity (Ho/He), and polymorphism information content (PIC) were calculated using GENALEX v. 6.5 [31]. Allelic richness (AR) in each population was assessed using the FSTAT program [32]. CERVUS v. 3.0.3 [33] was used to test for deviations from Hardy–Weinberg equilibrium (HWE) at each locus and for each population. Tests for linkage disequilibrium (LD) and the estimation of inbreeding coefficients (F_IS_) for each population were performed using GENEPOP v.4.0.1.1 [34]. A Bonferroni correction was applied to analyze deviation from HWE and for evidence of LD [35].

All of the *L. delicatula* DNA sequences were derived from the 183 samples obtained from eight different locations. Thirty-six sequences from 18 samples were downloaded from GenBank, including those from China, South Korea, and Japan (*ND2*: KC422353-KC422370; *ND6*: KC422371-KC422388) [10]. These sequences were aligned using CLUSTAL-X v 1.81 [36] and MEGA v. 6.0 [37]. The two mitochondrial fragments were concatenated for each sample. The number of haplotypes, haplotype diversity (Hd) [38], and nucleotide diversity were estimated by DnaSP v. 5.10 [39].

### 2.3. Haplotype Relationship Analyses

Two methods were used to reveal the relationships among haplotypes: a median joining network analysis and reconstruction of a phylogenetic tree. A median joining network analysis based on the haplotypes from 201 samples was performed in NETWORK v. 4.6.1.1 [40]. Three combined mitochondrial sequences from *Nilaparvata* (*Nilaparvata lugens*: LC461186; *Nilaparvata bakeri*: NC_033388; *Nilaparvata muiri*: NC_024627) and the haplotypes from 201 combined mitochondrial sequences of *L. delicatula* were used in phylogenetic analyses, performed using neighbor-joining (NJ), maximum likelihood (ML), and Bayesian inference (BI). The ModelFinder in PhyloSuite v.1.1.15 [41] was applied to find HKY + F + I model for ML and BI methods based on Akaike information criterion (AIC). The NJ and ML methods were carried out using MEGA 6 (with 1000 bootstraps) and IQ-TREE (with 5000 ultrafast bootstraps and 1000 replicates for the SH-aLRT branch test) [42], respectively. The BI method was applied using MrBayes v.3.2.1 [43]. Two separate analyses were run for 4 × 10^6^ generations with a sampling frequency of 100 generations. Additional generations were run until the average standard deviations of split frequencies were below 0.01, and effective sample size (ESS) was above 200. Finally, two separate analyses were run for 6 × 10^6^ generations, with the initial 25% sampled data discarded. The consensus tree and the posterior probability were obtained from the remaining sampled data.

### 2.4. Population Genetic Structure Analyses

Multiple methods were used to better understand the population genetic structure of *L. delicatula*. Initially, for the microsatellite dataset, POPTREE2 was used to construct an unrooted neighbor-joining (NJ) tree based on the genetic distances (*D_A_*) among populations [44,45]. The Bayesian inference-based program STRUCTURE v. 2.3.4 [46,47] was applied to detect clusters of multilocus genotypes in populations using admixture model and correlated allele frequencies. Twenty independent runs of each inferred cluster (K) from 1 to 8 were evaluated. One million Markov chain Monte Carlo (MCMC) repetitions with a 100,000 repetition burn-in period were included in each run. After running, STRUCTURE HARVESTER [48] was used to determine the most likely number of genetic clusters (K) under the method of the ad hoc statistic (ΔK) [49]. For the selected K, CLUMPP v1.1.2 [50] and DISTRUCT v. 1.1 [51] were adopted to permute the cluster labels across runs and display the genetic structure results. Population structure was confirmed using principle coordinate analysis (PCoA) implemented in GENALEX v. 6.5.

Next, ARLEQUIN v. 3.5.1.2 [52] was used to calculate the genetic differentiation (*F*_ST_) among sampling sites with 1000 permutations for both mitochondrial and microsatellite markers. Then, isolation by distance (IBD) was tested by the relationship of genetic values (*F*_ST_/(1-*F*_ST_)) and geographical distance (lnKm) at the population level for mtDNA and microsatellite datasets, implemented in the Mantel test [53] of IBDWS v. 3.23 [54,55]. The pairs for geographic distance matrix between populations were generated by Geographic Distance Matrix Generator v. 1.2.3 (http://biodiversityinformatics.amnh.org/opensource/gdmg/index.php). Finally, the distribution of genetic variance within and among the clusters for mtDNA and microsatellite datasets was conducted in ARLEQUIN v. 3.5.1.2 through the analysis of molecular variance (AMOVA) [52,56].

The migration rate (*M*) and the mutation-scaled population size (*θ*) was calculated by MIGRATE v.3.6.4 with full model [57]. The effective number of migrants of each population per generation (*Nem*) was *θM*. The first run was estimated from *F*_ST_, and the other three runs were started with *θ* and *M* from the previous run. In the Bayesian search strategy, one long chain with four independent replicates was conducted for each run with 1,000,000 generations (the first 10,000 were discarded as burn in). Heating chain was set: 1.0, 1.5, 3.0, and 1,000,000.

### 2.5. Historical Demography

BOTTLENECK v. 1.2.02 was used to detect the demographic history of population size variations [58,59]. Single step mutation of 95% and 10% multiple step mutation (a variance among multiple steps of 12) were set under three mutation models: the infinite allele model (IAM), the stepwise mutation model (SMM), and the two-phase mutation model (TPM). The significance of heterozygosity excess was evaluated using the Wilcoxon signed-rank test with 1000 simulation iterations [60].

The McDonald–Kreitman test (in DnaSP 5.10 [39]) was conducted on the mtDNA to rule out the possibility of a selective sweep. Tajima’s *D* [61] and Fu’s *Fs* [62] for eight Chinese populations were estimated with DnaSP 5.10.

## 3. Results

### 3.1. Genetic Diversity

Thirteen microsatellite markers for 183 individuals were successfully genotyped. Deviations from HWE were not detected in eight populations across different markers (P_HWE_ > 0.05) (Table S1). The results of Micro-checker showed no evidence of a null allele. Although mean null allele frequencies calculated using FreeNA across populations ranged from 0.002 to 0.048 (lower than 0.2), these results indicate little effect on the population genetic analyses. These markers were of middle to high polymorphism (PIC > 0.25) (Table S1) [63]. Therefore, all data from the 13 microsatellite markers were used to analyze the genetic diversity and population structure. The average Na and Ne per marker (Table 2) ranged 4.2 to 7.7 and from 2.55 to 4.19, respectively. Ar ranged from 3.94 to 7.23. He ranged 0.511 from 0.670, and Ho ranged between 0.536 and 0.675.

After final alignment, a total of 183 sequences were generated from eight populations with primers of *ND2* and *ND6* fragments, respectively. Seventeen *ND2* haplotypes and nine *ND6* haplotypes were uploaded to GenBank (accession numbers: MK450267–MK450288). In this study, 201 combined mitochondrial sequences were used to detect the genetic variation of mitochondrial DNA. The values of haplotype diversity, nucleotide diversity, and the average number of nucleotide differences of combined *ND2* and *ND6* were calculated in eight populations (Table 2).

### 3.2. Genetic Structure and Haplotype Relationship

The *F*_ST_ value (ranging from 0.0322 to 0.2583) based on microsatellite markers suggested a low to high level of genetic differentiation among populations (Table 3) [64]. The neighbor-joining tree based on *D_A_* revealed four lineages (Figure 1). Although ΔK had peaks in K = 2 and K = 4 (Figure S1), the most likely number of genetic clusters was four, which represented the uppermost level of genetic structure in China populations (Figure 1). Results of STRUCTURE corresponded to four lineages in the NJ tree. SD population (cluster 1) assigned to the first cluster differently from the other populations. BJ, HN, and AH populations (cluster 2) were assigned to the second cluster. SX and GS populations (cluster 3) remained as the third cluster. The individuals of the ZJ population assigned to the fourth cluster were similar to those of the FJ population (cluster 4). AMOVA analysis on the four clusters detected that the majority of genetic differentiation originated from differentiation within populations (84.25%, *p* < 0.0001) (Table 4). Significant differentiation was found among four clusters based on the microsatellite (8.64%, *p* < 0.0001) and the combined mitochondrial datasets (8.75%, *p* < 0.0001). The PCoA results also show a four-cluster pattern of genetic structure. The first two axes explain 40.94% and 27.11% of the overall variance on the population level (Figure S2). There is a clear pattern of IBD in *L. delicatula* based on the microsatellite dataset (Figure S3A: *r* = 0.378, *p* = 0.047).

**Figure 2:**
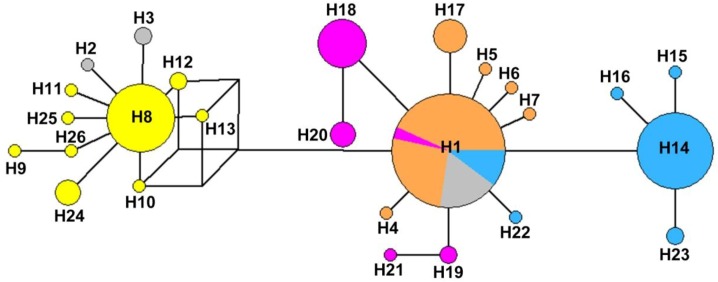
Haplotype network of *Lycorma delicatula* populations based on concatenated mitochondrial NADH dehydrogenase subunit 2 (*ND2*) and NADH dehydrogenase subunit 6 (*ND6*). Each circle represents a haplotype and sizes indicate the number of individuals. The haplotypes are identified by H1–H26. Except for H1, H8, and H14, the others are private haplotypes. The proportions of populations in haplotypes are indicated by the colors of circles. The colors are the same as the major clusters of populations inferred by STRUCTURE analysis when K = 4, except for gray (representing the haplotypes downloaded from GenBank).

**Figure 3:**
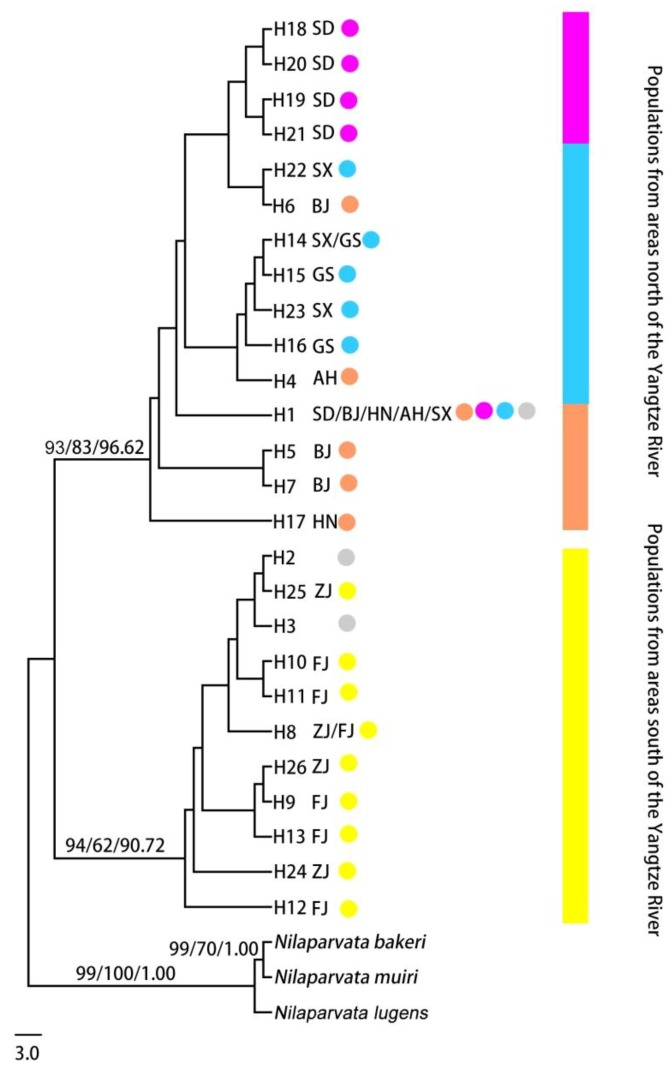
The phylogenetic tree of *Lycorma delicatula* populations in China, Korea, and Japan using neighbor-joining, maximum likelihood, and Bayesian inference. The haplotypes of Korea and Japan are H1; except for the haplotypes of Tiantai, Ningbo (H3) and Linan (H2), the other haplotypes of China are H1 [10]; the gray is used to represent these downloaded haplotypes. ZJ and FJ populations are in areas south of the Yangtze River; the other populations are included in areas north of the Yangtze River. The colors are the same as the major clusters of populations inferred by STRUCTURE analysis when K = 4. The bootstrap values of NJ and ML and the posterior probability of BI are given (>60).

The distribution of haplotypes is shown in Figure 2 and Table S2. Twenty-six haplotypes (H1–H26) were revealed in the 201 mitochondrial sequences. Three of them (H1, H8, and H14) were shared by different geographical populations, and the others were private haplotypes. It should be noted that the most common haplotype H1 was shared by 73 individuals in five geographical populations (except for the GS, ZJ, and FJ populations). The haplotype diversity and nucleotide diversity were higher in the SD, SX, and FJ populations than in the other populations. Furthermore, Chinese populations had more private haplotypes than did those from Korea (which had only one haplotype H1). In the phylogenetic tree for combined sequences (Figure 3), the haplotypes of Korea and Japan were close to the haplotypes of the populations south of the Yangtze River.

The phylogenetic relationships show that all haplotypes of populations were divided into two clusters: the haplotypes of populations north of the Yangtze River clustered closely, as well as the haplotypes of populations south of the Yangtze River (Figure 3). The *F*_ST_ values between populations ranged from 0.0001 to 0.9729 (Table 3). The private haplotypes (H15 and H16) from the GS population all clustered with haplotypes from the SX population, but one private haplotype (H22) from the SX population clustered with haplotypes from the SD and BJ populations. The AMOVA of the two clusters (the populations north of the Yangtze River: SD, BJ, HN, AH, SX, GS; the populations south of the Yangtze River: ZJ and FJ) revealed that most differentiation occurred within populations (90.60%, *p* < 0.0001) (Table S3), and there is a significant variance between two clusters based on the microsatellite (5.48%, *p* = 0.0293) and the combined mitochondrial datasets (4.63%, *p* = 0.0351). There was no significant IBD pattern between the genetic and geographical distances based on mtDNA (Figure S3B: *r* = 0.077, *p* = 0.698).

### 3.3. Genetic Connectivity

There are significant asymmetrical effective migrants of migrants between four clusters divided by the result of STRUCTURE analysis. The effective population size ranged from 0.0194 for cluster 1 to 0.0981 for cluster 2 (Table S4). The effective migrants leaving out of cluster 2 were highest with the values of 46.50; however, low values were detected for migrants entering into cluster 2 (10.02). The lowest number of migrants leaving out of cluster 1 was found with the values of 2.05, and the highest number of migrants entering into cluster 1 was detected with the value of 24.40. The effective migrants from populations south of the Yangtze River to populations north of the Yangtze River were 13.40 (based on microsatellite dataset) and 2.95 (based on mitochondrial dataset) (Table S5).

### 3.4. Historical Demography

Any result of a bottleneck was rejected for SX, GS, ZJ, and FJ populations under TPM (*p* > 0.05) (Table S6). All populations showed a normal L-shaped distribution. All populations showed a significant heterozygosity excess under IAM (*p* < 0.05), which may suggest a recent genetic bottleneck. Under TPM and SMM, BJ (TPM: *p* = 0.004; SMM: *p* = 0.009), HN (TPM: *p* = 0.027; SMM: *p* = 0.027), and AH (TPM: *p* = 0.003; SMM: *p* = 0.004) populations showed some evidence of heterozygosity excess, as well as the SD population under TPM (*p* = 0.021).

The McDonald–Kreitman neutrality test indicates no deviation from neutrality (McDonald–Kreitman’s test, *p* > 0.05), and the significantly negative values of Tajima’s *D* and Fu’s *Fs* suggests a recent demographic expansion for FJ populations (Tajima’s *D*: −1.909, *p* < 0.05; Fu’s *Fs*: −2.797, *p* = 0.043) (Table S6). BJ population showed an evidence of a recent demographic expansion with the significantly negative value of Fu’s *Fs* (Fu’s *Fs*: −3.021, *p* = 0.039).

## 4. Discussion

### 4.1. Genetic Diversity of L. delicatula Populations in China

Although the most common haplotype (H1) was found mostly in BJ, HN, and AH populations, both microsatellite and mtDNA datasets revealed a lower genetic diversity in these three populations than in other populations. This study also found the genetic diversity of *L. delicatula* populations in areas south of the Yangtze River to be higher than that in the north populations (Table 2) based on microsatellite and mtDNA datasets. The ZJ and FJ populations, by contrast, had a higher genetic diversity and more private haplotypes (Table 2, Table S2). The higher genetic diversity contributed to adapting to the environment and becoming common in the areas south of the Yangtze River. The SX population also had the same alleles and the same haplotype (H14) as exists in the GS population, and a close relationship was found between the SX and GS populations, which suggest that the GS population probably came from the SX population.

### 4.2. Population Genetic Structure and Historical Demography

The phylogenetic analysis (Figure 3) reveal a significant genetic differentiation between the populations south and north of the Yangtze River. Based on this analysis, as well as the previous study [10], all of the haplotypes are divided into two clades, i.e., the south and north clades of the Yangtze River. All populations south and north of the Yangtze River showed close relationships with the genetic distances for combined mitochondrial sequences being less than 1.6% between populations, which is below the criterion of 2% for insect species differentiation. Significant asymmetrical effective migrants between the cluster north of the Yangtze River and the cluster south of the Yangtze River was discovered by non-overlapping 95% confidence intervals (denoted in bold in Table S4). These close genetic relationships and asymmetrical migrants reveal that *L. delicatula* populations south of the Yangtze River may come from populations north of the Yangtze River. The haplotypes in Korea and Japan came from the north clade, which is consistent with previous suggestions [10].

The pairwise *F*_ST_ based on microsatellite and mtDNA datasets show a clear genetic differentiation and segregation among *L. delicatula* populations in China. There was a high level of genetic differentiation between SX/GS populations (cluster 3) and other populations, as well as ZJ/FJ (cluster 4) populations. These four populations have their private haplotypes and a higher genetic diversity, and all experienced a recent demographic expansion (*p* > 0.05 under TPM and SMM). Significant asymmetrical effective migrants from cluster 2 (BJ/HN/AH populations) to cluster 3 and cluster 4 were detected by non-overlapping 95% confidence intervals (denoted in bold in Table S5). This suggests that the BJ, HN, and AH populations were the source populations that migrated to the other populations. *L. delicatula* might have shifted their ranges from BJ, HN, and AH populations into populations in to more extreme climate regions, including SX/GS populations in the colder regions of northwest China and ZJ/FJ populations in the warmer regions south of the Yangtze River.

Six populations of *L. delicatula* in the areas north of the Yangtze River were subdivided further into three clusters by using microsatellite markers, and they contained three common haplotypes (H1, H18, and H14) using mitochondrial markers. *L. delicatula* was previously known to occur in only Shaanxi, Shandong, and Hebei in the 1930s [10,25]. Now there is a high level of genetic differentiation between six populations based on the microsatellite dataset. The BJ, HN, and AH populations were regarded as the major source of migrants with the high number of migrants moving out and the low number of migrants entering. It can be stated that *L. delicatula* spread from the BJ, HN, and AH populations to the other populations in the areas north of the Yangtze River. The phylogenetic relationship of private haplotypes from the GS (H15 and H16), SX (H22), BJ (H6), SD (H18-21), HN (H4), and AH (H17) populations and asymmetrical migrants between clusters reveal that the potential gene flow occurred between six populations.

Compared with the mitochondrial dataset, the microsatellite dataset of the *L. delicatula* populations in China shows a significant IBD pattern, further subdivision of genetic structure, and more asymmetrical effective migrants. It means microsatellite markers can provide more information than mitochondrial fragments on population structure, population dynamics, and dispersal patterns. These two markers showed some migrants between different populations. A probable cause is the increase in afforested areas in recent years, which in turn expanded the distribution of the host plants throughout China and increased migrants between populations. Meanwhile, higher genetic diversity and some biological features (including the habitat for overwintering by eggs, polyphagy, and high fecundity) all contributed to the successful establishment of *L. delicatula* in new areas [16,20,24]. In addition, the geographical distances, geographical barrier (such as the Yangtze River and Qinling Mountains), and diverse climates likewise play a major role in the gene flow and the high level of genetic differentiation between populations with high genetic diversity.

## 5. Conclusions

A significant genetic differentiation was found among Chinese *L. delicatula* populations. There was a high level of genetic differentiation between SX/GS populations and other populations, as well as ZJ/FJ populations. Significant asymmetrical effective migrants from BJ/HN/AH populations to SX/GS populations and ZJ/FJ populations were detected. BJ/HN/AH populations were regarded as the major producer of migrants to the other populations. This study improves our understanding of the origin and the dispersal of *L. delicatula*. Biological features, together with numerous suitable habitats, may account for this establishment of *L. delicatula* in new areas. In the recent past, sudden outbreaks of this pest occurred in China, Korea, and Japan, and this pest has also invaded into North America. Future work should pay more attention to the possible invasion pattern of *L. delicatula* based on microsatellite and mitochondrial markers.

## Figures and Tables

**Table 1:** Collecting information for *Lycorma delicatula*.

Collecting Locality		Code of Specimens	Latitude (N)/Longitude (E)	Collecting Dates
North China	Weihai, Shandong	SD	37.50/122.12	October 2016
	Beijing	BJ	39.96/116.33	October 2016
	Anyang, Henan	HN	36.10/114.39	October 2016
	Yangling, Shaanxi	SX	34.25/108.08	October 2016
	Lanzhou, Gansu	GS	36.10/103.71	August 2017
South China	Hefei, Anhui	AH	31.86/117.25	July 2017
	Quzhou, Zhejiang	ZJ	29.25/118.11	August 2017
	Shanming, Fujian	FJ	26.26/117.63	July 2017

**Table 2:** The genetic diversity of *L. delicatula* populations based on 13 microsatellite markers and two mtDNA markers. Pop, population label; n, no. samples; N_a_, mean number of alleles per marker; N_e_, mean effective number of alleles per marker; Ar, allelic richness; Ho, observed heterozygosity; He, expected heterozygosity; Nh, no. haplotypes; Hd, haplotype diversity; π, nucleotide diversity; k, average number of nucleotide differences. Thirty-six mitochondrial sequences from China, South Korea, and Japan populations were downloaded from GenBank (*ND2*: KC422353–KC422370; *ND6*: KC422371–KC422388).

Pop	Microsatellite Markers						mtDNA				
	n	Na	Ne	Ar	Ho	He	n	Nh	Hd	π (%)	k
SD	24	4.2	2.56	3.94	0.536	0.511	24	4	0.634	0.112	0.993
BJ	24	5.2	2.78	4.57	0.561	0.557	24	4	0.239	0.028	0.250
HN	25	5.5	2.55	4.75	0.525	0.547	24	2	0.431	0.048	0.431
AH	24	4.4	2.39	4.03	0.587	0.565	24	2	0.083	0.009	0.083
SX	24	6.8	3.79	6.03	0.612	0.623	24	3	0.627	0.084	0.750
GS	24	5.9	3.33	5.42	0.630	0.643	24	2	0.163	0.019	0.167
ZJ	22	7.7	4.19	6.99	0.675	0.670	22	3	0.455	0.093	0.494
FJ	16	7.2	3.91	7.23	0.572	0.628	17	4	0.588	0.104	0.926
China							7	3	0.667	0.728	6.476
Japan							1	1			
South Korea							10	1			

**Table 3:** Pairwise estimates of *F*_ST_ based on 13 microsatellite markers (below diagonal) and two mtDNA markers (above diagonal). All pairwise comparisons of *F*_ST_ values are statistically significant.

Populations	SD	BJ	HN	AH	SX	GS	ZJ	FJ
SD	0.0000	0.4858	0.4822	0.5217	0.5137	0.7320	0.9336	0.9125
BJ	0.2583	0.0000	0.1826	0.0001	0.4000	0.8276	0.9648	0.9473
HN	0.1578	0.1243	0.0000	0.2283	0.4094	0.7826	0.9564	0.9378
AH	0.1610	0.1272	0.0419	0.0000	0.4444	0.8889	0.9729	0.9567
SX	0.1819	0.1277	0.1048	0.1152	0.0000	0.2667	0.9429	0.9227
GS	0.1998	0.1443	0.1359	0.1419	0.0701	0.0000	0.9716	0.9564
ZJ	0.1577	0.2064	0.1577	0.1460	0.0856	0.1144	0.0000	0.0435
FJ	0.1910	0.2218	0.1791	0.1623	0.0919	0.1559	0.0322	0.0000

**Table 4:** AMOVA results of eight *L. delicatula* populations between four clusters inferred from STRUCTURE analysis based on microsatellite and mtDNA datasets.

Markers	Source of Variation	d.f.	Sum of Squares	Variance Components	Percentage Variation	Fixation Indices
Microsatellite markers	Among clusters	3	156.846	0.3895 Va	8.64	FCT = 0.0864
						*p* < 0.0001
	Among populations within g clusters	4	73.649	0.3205 Vb	7.11	FSC = 0.0778
						*p* < 0.0001
	Within populations	358	1359.953	3.7988 Vc	84.25	FST = 0.1575
						*p* < 0.0001
mtDNA	Among clusters	3	43.344	0.24768 Va	8.75	FCT = 0.9125
						*p* < 0.0001
	Among populations within clusters	4	4.339	0.03672 Vb	1.30	FSC = 0.1291
						*p* < 0.0001
	Within populations	175	355.815	2.45459 Vc	89.95	FST = 0.8995
						*p* < 0.0001

## Supplementary Materials

The following are available online at https://www.mdpi.com/2075-4450/10/10/312/s1: Table S1: Genetic polymorphism of 13 microsatellite markers and HWE tests in eight populations of *L. delicatula.* Table S2: Distribution of *ND2* and *ND6* shared haplotypes in *Lycorma delicatula* populations. Table S3: AMOVA results of eight *L. delicatula* populations between two clusters (the cluster north of the Yangtze River and the cluster south of Yangtze River). Table S4: The population size and numbers of effective immigrants per generation between four clusters inferred from STRUCTURE analysis based on the microsatellite and the combined mitochondrial datasets. Table S5: The population size and numbers of effective immigrants per generation between two clusters (the cluster north of the Yangtze River and the cluster south of Yangtze River) based on the microsatellite and the combined mitochondrial datasets. Table S6: The Wilcoxon test under TPM and SMM models based on the microsatellite datasets and Tajima’s *D* and Fu’s *Fs* tests based on the combined mitochondrial dataset. Figure S1: Estimated number of genetic clusters obtained with STRUCTURE analysis for K ranging from one to eight using 13 microsatellite markers for eight populations. Figure S2: Results of principle coordinate analysis based on 13 microsatellite markers in eight *Lycorma delicatula* populations in China. Figure S3: Scatter plots of the genetic distance isolation by geographical distance among *Lycorma delicatula* populations in China based on 13 microsatellite markers and two mitochondrial markers.
