# Supplementary material for: Genetic Diversity and Population Structure of Natural Lycorma delicatula (White) (Hemiptera: Fulgoridea) Populations in China as Revealed by Microsatellite and Mitochondrial Markers
Source: Insects. 2019 Sep 23;10(10):312. doi: 10.3390/insects10100312 (PMC6835943; doi:10.3390/insects10100312)
Supplement: Supplementary file 1 [file insects-10-00312-s001.pdf]

Genetic diversity and population structure of natural *Lycorma delicatula* (White) (Hemiptera: Fulgoridea) populations in China as revealed by microsatellite and mitochondrial markers

Li Zhang[1], Wenhui Zhao[2], Fuping Wang[3], Daozheng Qin[1]*

[1] Key Laboratory of Plant Protection Resources and Pest Management of the Ministry of Education; Entomological Museum, Northwest A&F University, Yangling, Shaanxi 712100, China; zhangli0927@aliyun.com

[2] Plant Quarantine Station of Plant Protection, Kaifeng, Henan 475000, China; zhaowenhui15@163.com

[3] Yangling Xianglin Agriculture Biotechnology Company Limited, Yangling, Shaanxi 712100, China; fpw616@126.com

*Correspondence：Dr. Dao-Zheng Qin; qindaozh0426@aliyun.com; Tel: 86-29-87092524; Postal Address: Key Laboratory of Plant Protection Resources and Pest Management of the Ministry of Education; Entomological Museum, Northwest A&F University, Yangling, Shaanxi 712100, China

Supplementary Tables & Figures

Table S1. Genetic polymorphism of 13 microsatellite markers and HWE test in eight populations of *Lycorma delicatula*. K: Observed number of alleles; PIC: Polymorphism information content; Fnull: Frequency of null allele. P_{HWE}: P-values of Hardy-Weinberg equilibrium test

Microsatellite markers<br>GenBank accession no.<br>Annealing temperature (℃)		SD	BJ	HN	AH	SX	GS	ZJ	FJ	Mean
LD-D1	K	4	6	9	7	7	7	5	8	6.6
JF913272	PIC	0.456	0.721	0.758	0.681	0.704	0.677	0.649	0.695	0.668
60	Fnull	0.000	0.021	0.030	0.000	0.033	0.055	0.000	0.096	0.029
	P_{HWE}	0.974	0.433	0.869	0.856	0.120	0.734	0.528	0.315	
LD-D4	K	2	7	7	6	10	12	14	12	8.8
JF913275	PIC	0.337	0.633	0.735	0.667	0.786	0.753	0.781	0.78	0.684
60	Fnull	0.000	0.000	0.016	0.079	0.000	0.042	0.174	0.012	0.040
	P_{HWE}	0.260	0.950	0.465	0.661	0.431	0.272	0.236	0.424	
LD-D5	K	7	16	12	9	20	13	17	16	13.8
JF913276	PIC	0.595	0.858	0.73	0.642	0.905	0.869	0.874	0.908	0.798
55	Fnull	0.035	0.008	0.084	0.083	0.112	0.000	0.059	0.000	0.048
	P_{HWE}	0.988	0.210	0.060	0.100	0.300	0.517	0.074	0.214	
Lde01	K	2	4	3	2	2	4	2	3	2.8
HQ644424	PIC	0.375	0.187	0.285	0.337	0.305	0.365	0.362	0.294	0.314

56	Fnull	0.000	0.000	0.007	0.042	0.000	0.000	0.000	0.000	0.006
	P_{HWE}	0.414	0.999	0.962	0.533	0.586	0.908	0.248	0.837	
Lde03	K	3	3	2	3	4	3	5	5	3.5
HQ644426	PIC	0.264	0.361	0.189	0.391	0.402	0.405	0.51	0.483	0.376
56	Fnull	0.045	0.000	0.000	0.000	0.000	0.118	0.000	0.024	0.023
	P_{HWE}	0.658	0.925	0.495	0.112	0.602	0.209	0.708	0.210	
Lde04	K	2	4	4	4	5	4	6	4	4.1
HQ644427	PIC	0.146	0.503	0.429	0.615	0.479	0.611	0.406	0.262	0.431
54	Fnull	0.000	0.027	0.000	0.000	0.000	0.000	0.000	0.103	0.016
	P_{HWE}	0.648	0.740	0.597	0.431	0.380	0.899	0.999	0.368	
Lde05	K	3	7	7	3	5	5	7	9	5.8
HQ644428	PIC	0.499	0.711	0.593	0.496	0.657	0.626	0.71	0.746	0.630
55	Fnull	0.051	0.000	0.000	0.000	0.000	0.053	0.092	0.043	0.030
	P_{HWE}	0.418	0.479	0.997	0.397	0.240	0.666	0.773	0.059	
Lde06	K	2	3	4	4	4	3	6	5	3.9
HQ644429	PIC	0.258	0.399	0.42	0.465	0.518	0.531	0.584	0.4	0.447
55	Fnull	0.000	0.000	0.000	0.000	0.000	0.012	0.000	0.000	0.002
	P_{HWE}	0.258	0.565	0.895	0.057	0.873	0.528	0.286	0.906	
Lde07	K	10	6	8	5	10	8	11	12	8.8
HQ644430	PIC	0.851	0.365	0.61	0.461	0.84	0.785	0.842	0.827	0.698
57	Fnull	0.022	0.018	0.000	0.000	0.020	0.067	0.000	0.016	0.018
	P_{HWE}	0.092	0.999	0.118	0.898	0.375	0.072	0.103	0.089	
Lde08	K	6	3	3	2	4	6	5	4	4.1
HQ644431	PIC	0.75	0.378	0.364	0.375	0.478	0.649	0.483	0.472	0.494
56	Fnull	0.000	0.006	0.000	0.138	0.000	0.000	0.088	0.000	0.029
	P_{HWE}	0.120	0.899	0.901	0.420	0.670	0.872	0.400	0.769	
Lde09	K	4	2	4	3	5	4	3	3	3.5
HQ644432	PIC	0.507	0.275	0.33	0.301	0.155	0.479	0.436	0.416	0.362
57	Fnull	0.022	0.000	0.005	0.000	0.000	0.000	0.000	0.104	0.016
	P_{HWE}	0.689	0.197	0.987	0.164	0.999	0.230	0.966	0.238	
Lde11	K	6	2	6	7	9	5	16	10	7.6
HQ644434	PIC	0.547	0.371	0.491	0.456	0.795	0.546	0.91	0.848	0.621
56	Fnull	0.010	0.023	0.113	0.000	0.000	0.000	0.009	0.027	0.023
	P_{HWE}	0.875	0.736	0.10	0.839	0.857	0.168	0.495	0.092	

Lde13	K	4	5	3	3	4	3	3	3	3.5
HQ644436	PIC	0.242	0.571	0.404	0.441	0.387	0.264	0.415	0.401	0.391
55	Fnull	0.000	0.035	0.000	0.000	0.000	0.000	0.000	0.020	0.007
	P_{HWE}	0.995	0.308	0.737	0.506	0.112	0.811	0.468	0.499	

Table S2. Distribution of ND2 and ND6 shared haplotypes in *Lycorma delicatula* populations. Eighteen combined mitochondrial sequences from China (7 sequences), South Korea (10 sequences) and Japan (1 sequences) populations were downloaded from GenBank.

Haplotypes	SD	BJ	HN	AH	SX	GS	ZJ	FJ	China	South Korea	Japan	Total
H1	3	21	17	23	9				4	10	1	88
H2									1			1
H3									2			2
H4				1								1
H5		1										1
H6		1										1
H7		1										1
H8							16	11				27
H9								1				1
H10								1				1
H11								1				1
H12								2				2
H13								1				1
H14					12	22						34
H15						1						1
H16						1						1
H17			7									7
H18	14											14
H19	2											2
H20	4											4
H21	1											1
H22					1							1
H23					2							2
H24							4					4

H25	1	1
H26	1	1

Table S3. AMOVA results of eight *L. delicatula* populations between two clusters (the cluster north of the Yangtze River and the cluster south of Yangtze River).

	Source of variation	d.f.	Sum of squares	Variance components	Percentage variation	Fixation indices
Microsatellite markers	Among clusters	1	56.320	0.2516 Va	5.48	FCT = 0.0548 P = 0.0293
	Among populations within clusters	6	174.175	0.5432 Vb	11.82	FSC = 0.1251 P < 0.0001
	Within populations	358	1359.953	3.7988 Vc	82.70	FST = 0.1730 P < 0.0001
mtDNA	Among clusters	1	43.344	0.2477 Va	4.63	FCT = 0.9537 P = 0.0351
	Among populations within clusters	6	36.998	0.2552 Vb	4.77	FSC = 0.5074 P < 0.0001
	Within populations	175	303.155	4.8491 Vc	90.60	FST = 0.9061 P < 0.0001

Table S4. The population size and numbers of effective immigrants per generation between four clusters inferred from STRUCTURE analysis based on the microsatellite and the combined mitochondrial datasets. θ: the mutation-scaled population size; *Nem*: effective number of migrants per generation; Total in: effective number of migrants entering into each cluster per generation; Total out: effective number of migrants leaving out of each cluster per generation.

	**Clusters**	***θ***	***Nem***					
			**1→**	**2→**	**3→**	**4→**	**Total in**	**Total out**
Microsatellite markers	**1**	0.0194	-	**10.42**	**8.27**	5.71	24.40	2.05
	**2**	0.0981	0.50	-	5.51	4.01	10.02	46.50
	**3**	0.0974	0.67	**27.94**	-	7.66	36.27	21.58
	**4**	0.0967	0.88	**8.14**	7.80	-	16.82	17.38
mtDNA	**1**	0.0151	-	**9.26**	**7.16**	1.85	18.27	13.17
	**2**	0.0138	4.10	-	4.71	1.85	10.66	19.14

	**3**	0.0156	7.61	**8.53**	-	2.15	18.29	13.43
	**4**	0.0196	1.46	1.35	1.56	-	4.37	5.85

Table S5. The population size and numbers of effective immigrants per generation between two clusters (the cluster north of the Yangtze River and the cluster south of Yangtze River) based on the microsatellite and the combined mitochondrial datasets. θ: the mutation-scaled population size; *Nem*: effective number of migrants per generation; Total in: effective number of migrants entering into each cluster per generation; Total out: effective number of migrants leaving out of each cluster per generation.

	**Clusters**	***θ***	***Nem***	
			**North→**	**South→**
Microsatellite markers	**North**	0.0983	-	9.59
	**South**	0.0982	**13.40**	
mtDNA	**North**	0.0151	-	1.22
	**South**	0.0138	2.95	-

Table S6. The Wilcoxon test under TPM and SMM models based on the microsatellite datasets and Tajima's D and Fu's Fs tests based on the combined mitochondrial dataset.

Pop	TPM	SMM	Tajima's D	Fu's Fs
SD	0.021	0.068	-0.200	-0.905
BJ	0.004	0.009	-1.733	-3.021
HN	0.027	0.027	1.027	1.230
AH	0.003	0.004	-1.159	-1.028
SX	0.244	0.455	-0.167	-0.484
GS	0.068	0.127	-1.515	-2.078
ZJ	0.216	0.244	-1.035	-1.506
FJ	0.068	0.191	-1.909	-2.797

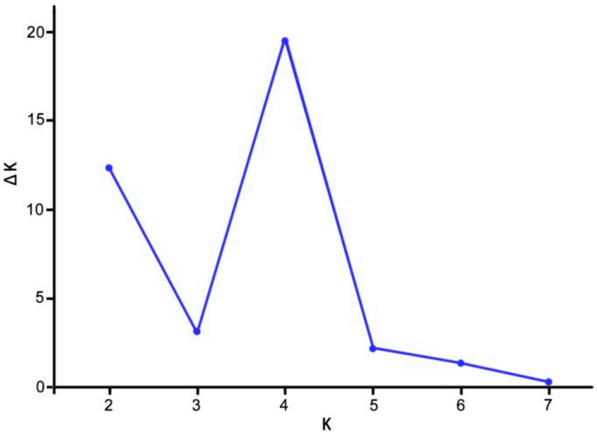

Figure S1. Estimated number of genetic clusters obtained with STRUCTURE analysis for K ranging from one to eight using 13 microsatellite markers for eight populations. The most likely number of genetic cluster (K) was four.

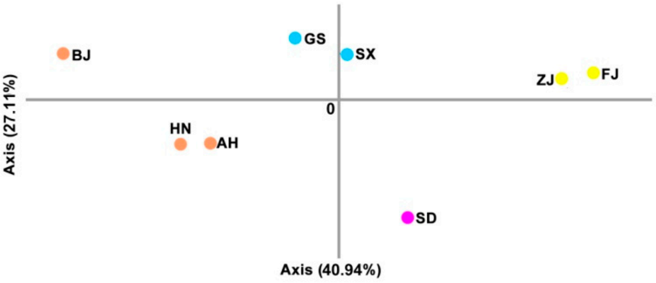

Figure S2. Results of principle coordinate analysis based on 13 microsatellite markers in eight *Lycorma delicatula* populations in China. Each label represents a population. The colors indicate the major cluster inferred by STRUCTURE analysis.

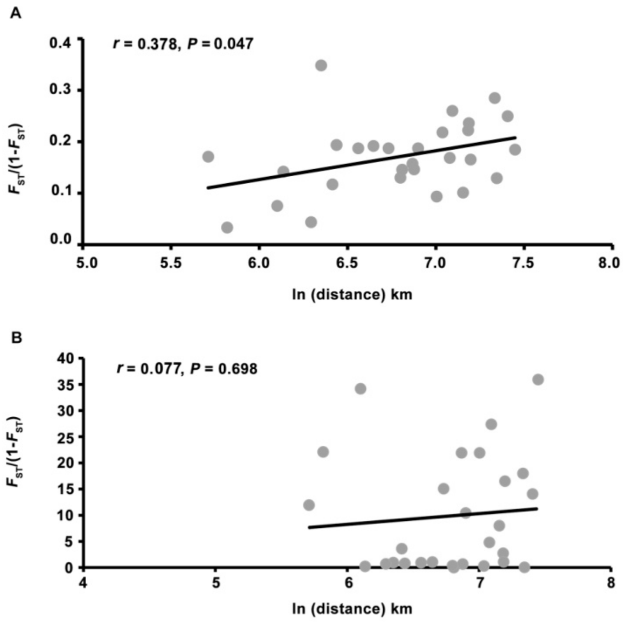

Figure S3. Scatter plots of the genetic distance isolation by geographical distance among *Lycorma delicatula* populations in China based on 13 microsatellite markers and two mitochondrial markers. (A) and (B) are based on microsatellite and mitochondrial markers, respectively.
